# Supplementary material for: Single cell transcriptomics of primate sensory neurons identifies cell types associated with chronic pain
Source: Nat Commun. 2021 Mar 8;12:1510. doi: 10.1038/s41467-021-21725-z (PMC7940623; doi:10.1038/s41467-021-21725-z)
Supplement: Supplementary file 1 — Supplementary Information [file 41467_2021_21725_MOESM1_ESM.pdf]

Supplementary Information

Supplementary Figs.

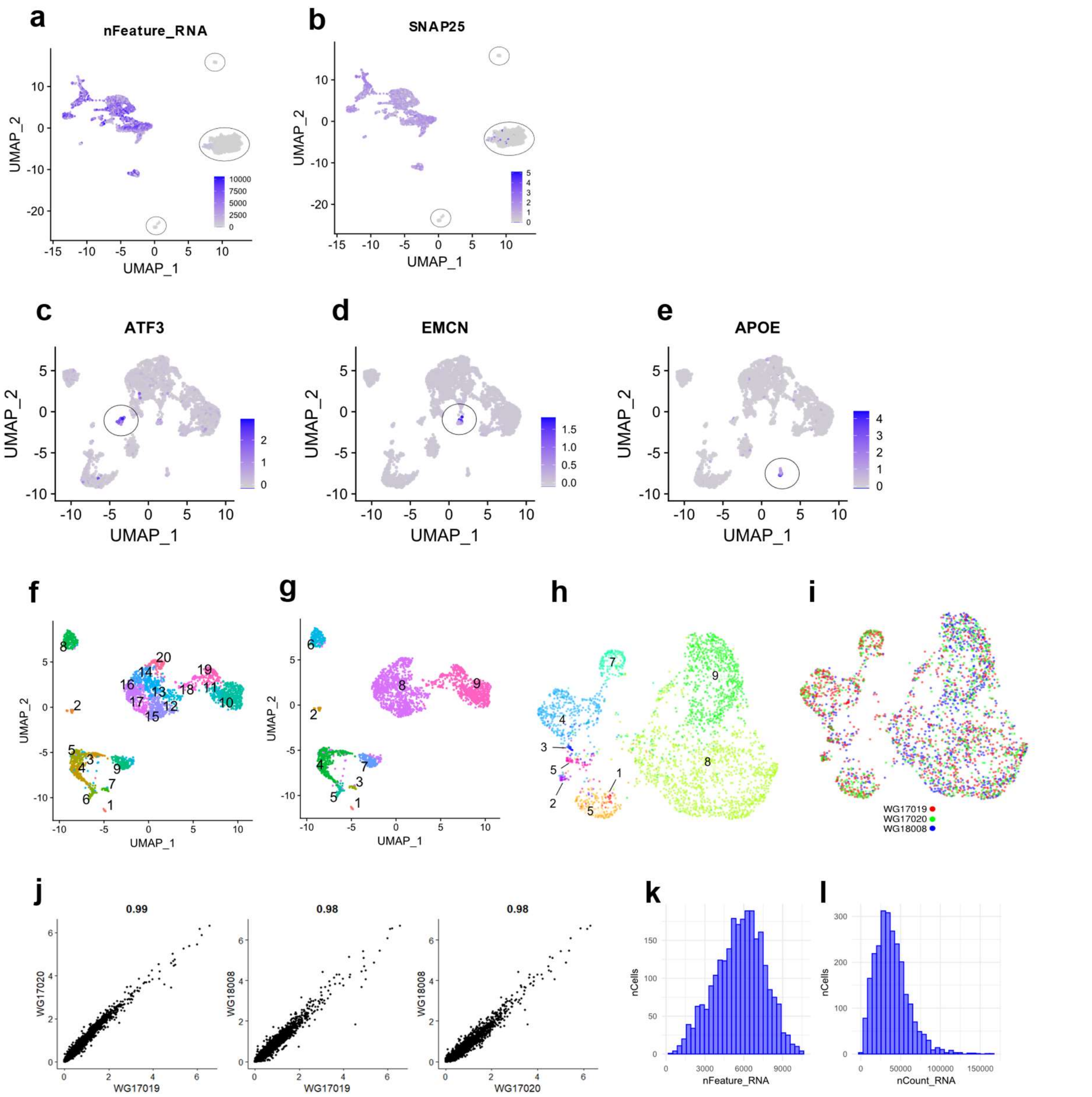

Supplementary Fig. 1. Clustering of macaque STRT-2i-seq data. (a, b) UMAPs showing removed clusters (circled) based on low gene detection (a) and lack of neuronal identity (b). (c-e) UMAPs showing removed clusters (circled) based on injury (c) non-neuronal (d) and satellite-glia (e) markers. Genes shown are representative of several marker genes. (f) UMAP showing the final 2,518 neurons after over-clustering. (g) UMAP showing the final nine neuron clusters after merging highly similar types. (h-i) UMAPs showing clustering results using an alternative method (Conos). (h) Conos clusters with numbering from original analysis showing near perfect match. (i) UMAP showing the equal distribution of cells from different individuals among the Conos clusters. (j) Scatterplots showing linear correlation of inter-individual gene expression. The Pearson correlation coefficient is shown above each plot. (k-l) Histograms showing distribution of (k) detected genes and (l) UMIs in the STRT-2i-seq data (n=3).

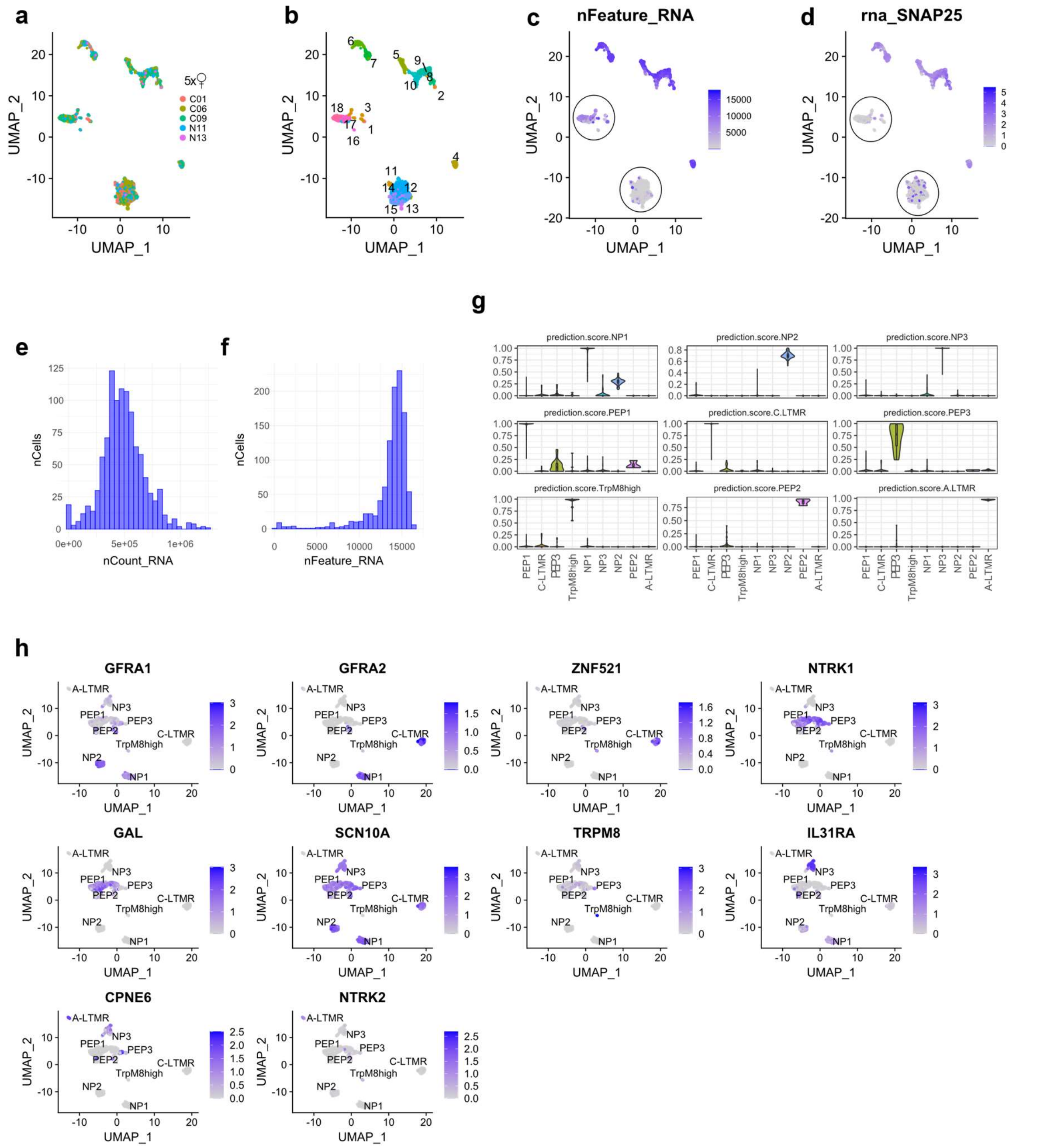

Supplementary Fig. 2. Analysis of macaque Smart-seq2 data. (a) UMAP distribution of cells originating from the individual animals after clustering. (b) UMAP showing numeric labels after primary clustering. (c, d) UMAPs showing removed clusters based on (c) low gene detection and (d) lack of neuronal markers (circled clusters). (e, f) Histograms showing the (e) transcript count and (f) detected gene count distribution in the Smart-seq2 data (n=5). (g) Violin plots showing the prediction scores from the label transfer between the STRT-2i-seq and Smart-seq2 data. Boxplot defines the median, interquartile range (IQR) and 1.5 * IQR (whiskers). (h) UMAPs showing mouse canonical marker gene expression in the Smart-seq2 macaque clusters after label transfer.

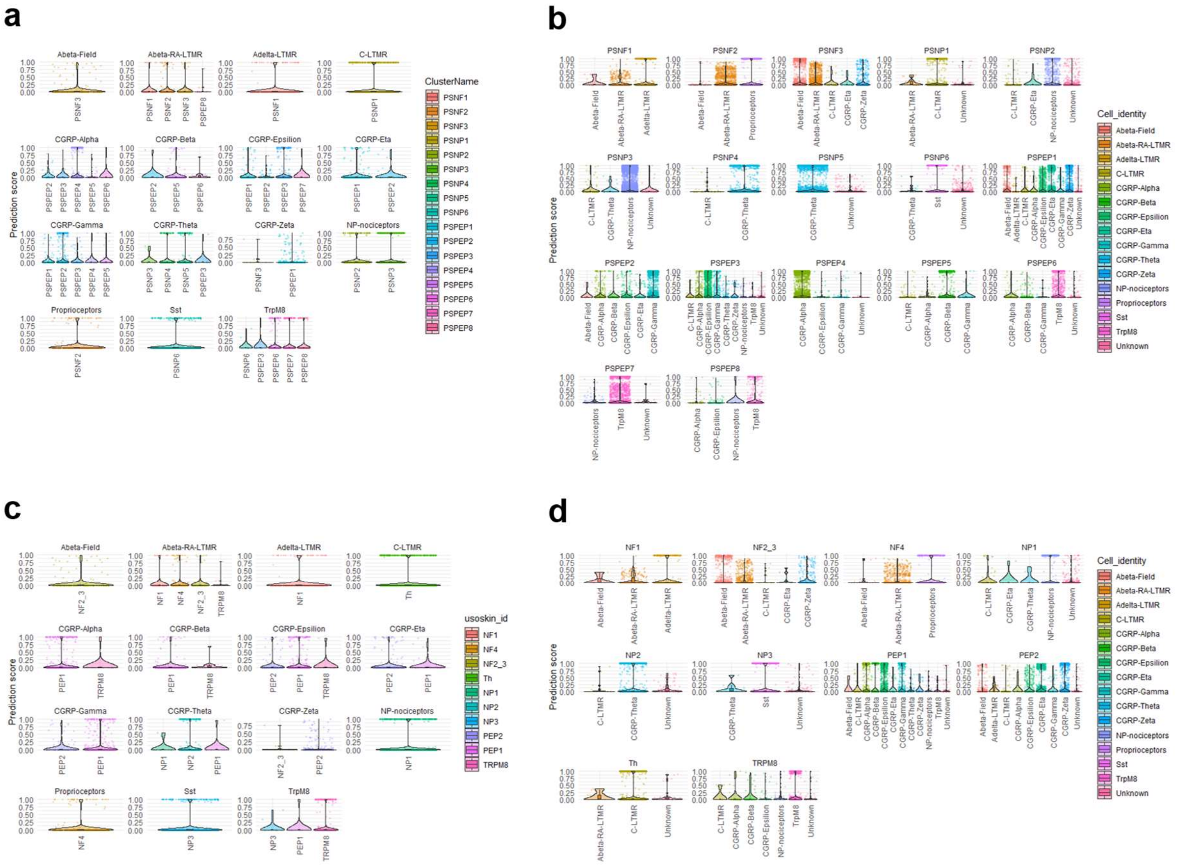

Supplementary Fig. 3. Prediction scores from label transfer between Zeisel et al. and Sharma et al. data. (a, b) Violin plots showing prediction scores from the label transfer from (a) Sharma to Zeisel and (b) Zeisel to Sharma. (c, d) Violin plots showing prediction scores from the label transfer from (c) Sharma to Zeisel and (d) Zeisel to Sharma when using Usoskin et al., 2015 nomenclature.

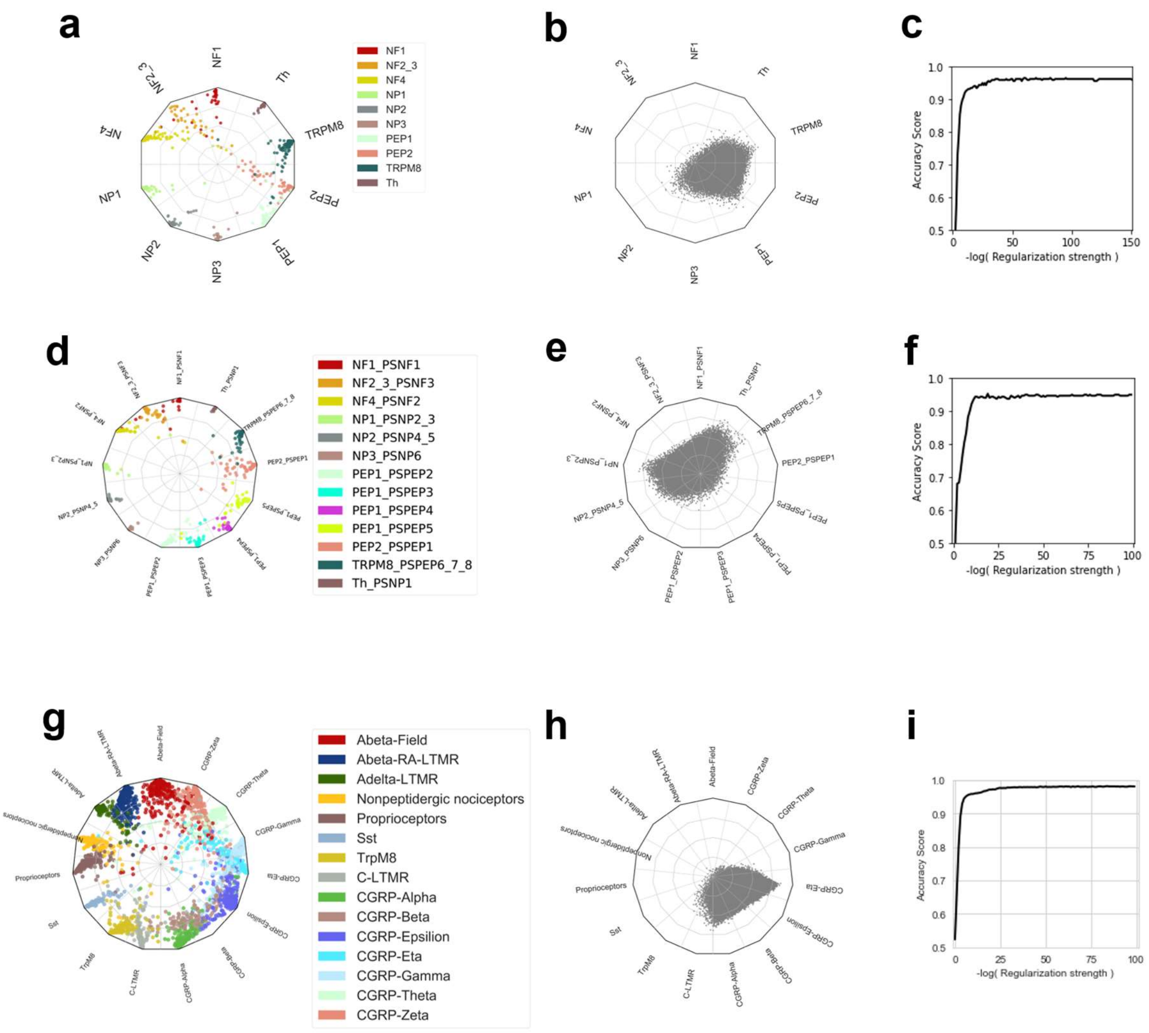

Supplementary Fig. 4. Scoring modules for prediction of sensory neuron types. (a) A radar-plot showing cell-type fractions of the reference mouse DRG neurons (1/3 of cells from Zeisel) from the neural-network scoring module as described in Methods section. Each dot represents one cell, and the color coding is based on unique cell clusters. (b) Negative control obtained from the same module after random permutation of the features in the training dataset and visualized in the radar plot. (c) Accuracy score for the learning module. (d-f) Scoring module from Zeisel data using original Zeisel nomenclature. (g-i) Scoring module from Sharma data using original Sharma nomenclature.

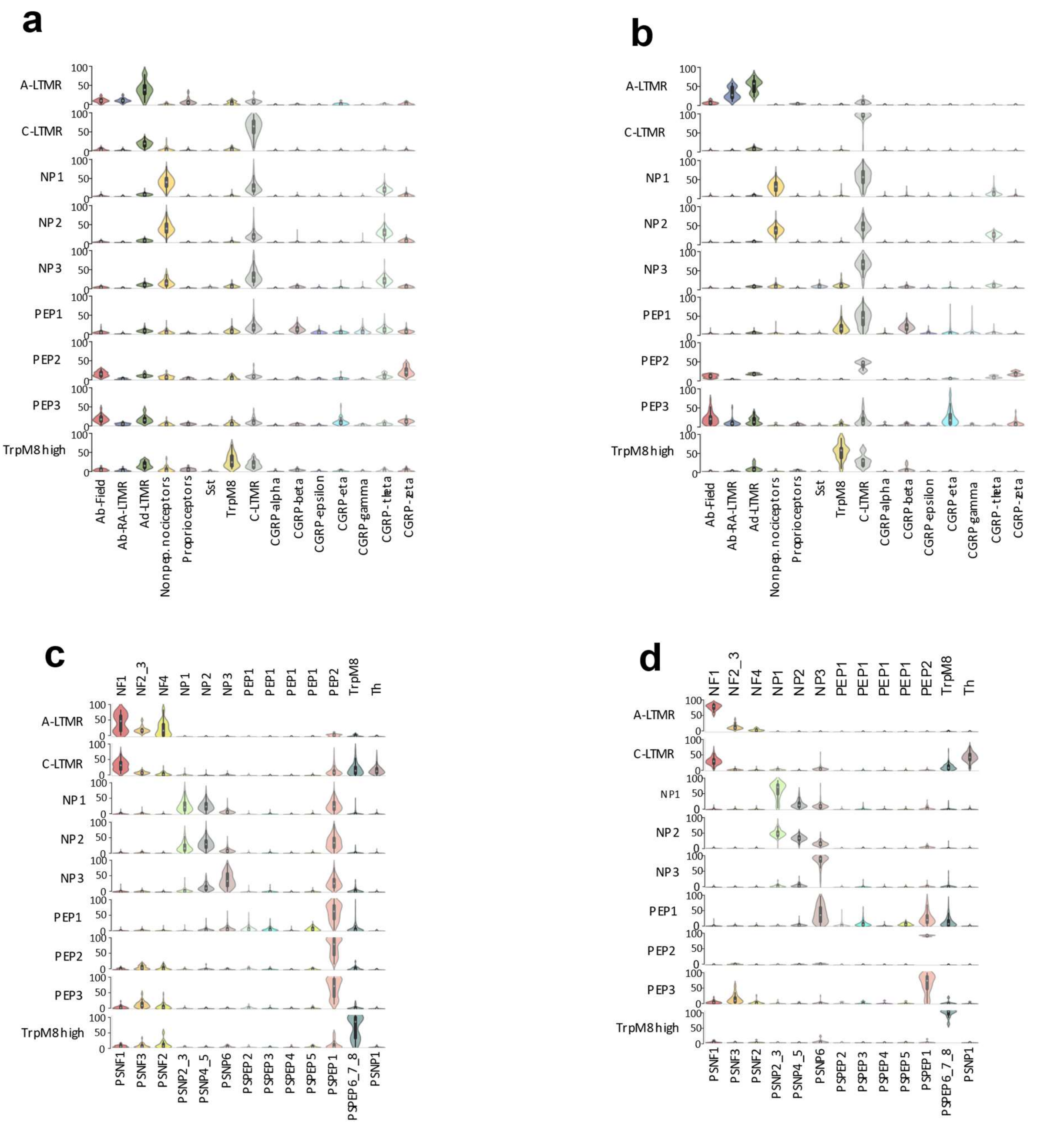

Supplementary Fig. 5. Prediction of macaque DRG neuron types from mouse data. Violin plots showing prediction probability scores between (a) macaque types (y-axis, STRT-2i-seq, n=3) and Sharma mouse types (x-axis), (b) macaque types (y-axis, Smart-seq2, n=5) and Sharma mouse types (x-axis), (c) macaque types (y-axis, STRT-2i-seq, n=3) and Zeisel mouse types (x-axis), (d) macaque types (y-axis, Smart-seq2, n=5) and Zeisel mouse types. (c) and (d) have Zeisel and Usoskin nomenclatures on bottom and top, respectively. Boxplots inside violins define the median, interquartile range (IQR) and 1.5 * IQR (whiskers).

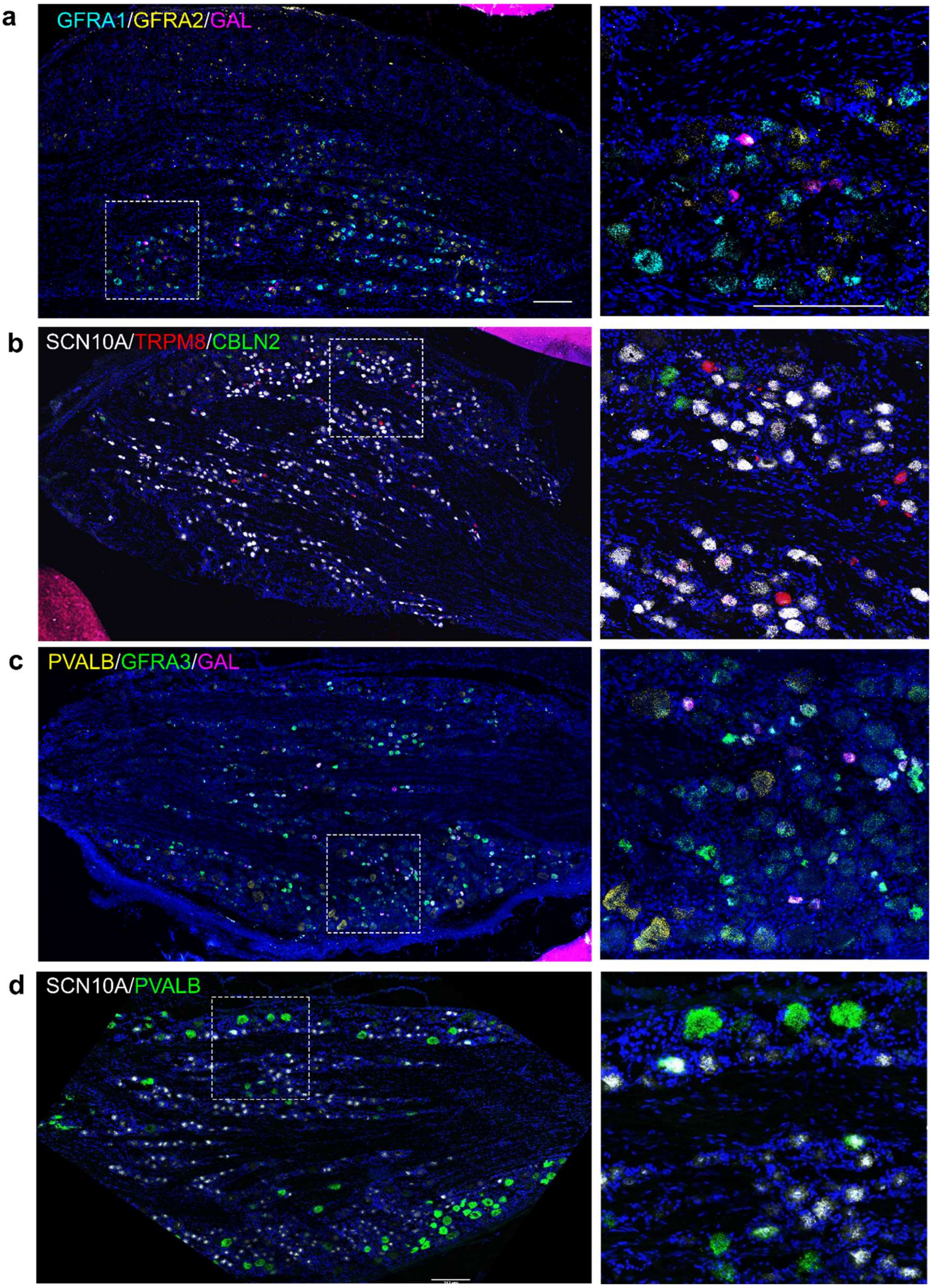

Supplementary Fig. 6. Validation of macaque clusters by triple in situ hybridization. (a-d) Images of entire macaque DRG sections after performing *in situ* hybridization using probes specific for either (a) GFRA1 (light blue), GFRA2 (yellow), and GAL (purple), (b) SCN10A (white), TRPM8 (red), and CBLN2 (green), (c) PVALB (yellow), GFRA3 (green), and GAL (purple) or (d) SCN10A (white) and PVALB (green). The white hatched box on the left images is shown at higher magnification on the right side of each panel. Scale bar=250 µm. The experiments were repeated independently with similar results in two animals (n=2).

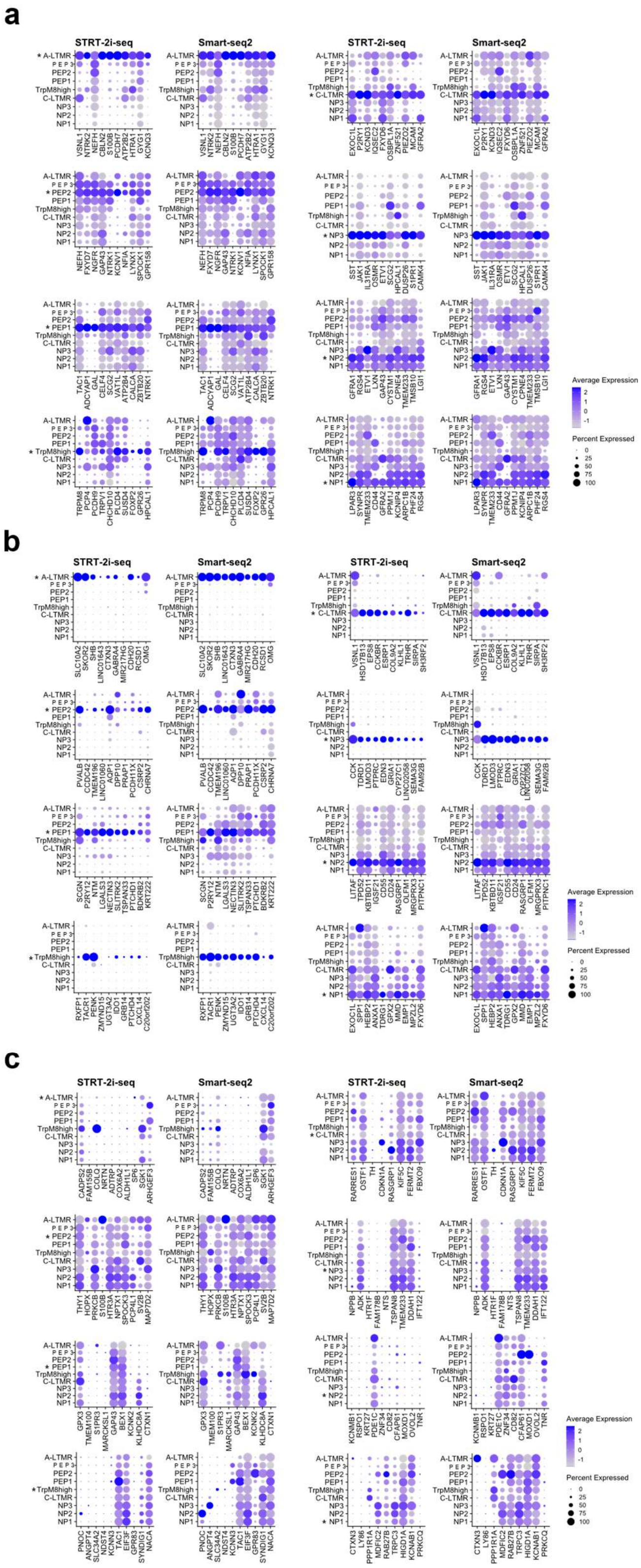

Supplementary Fig. 7. Side-to-side gene expression comparison between STRT-2i-seq and Smart-seq2 data. (a) Top genes shared between corresponding cell types of macaque and mouse plotted in both macaque datasets. (b) Top macaque enriched genes plotted in both macaque datasets. (c) Top mouse enriched genes plotted in both macaque datasets. Cell type to compare is marked with an asterisk in each panel.

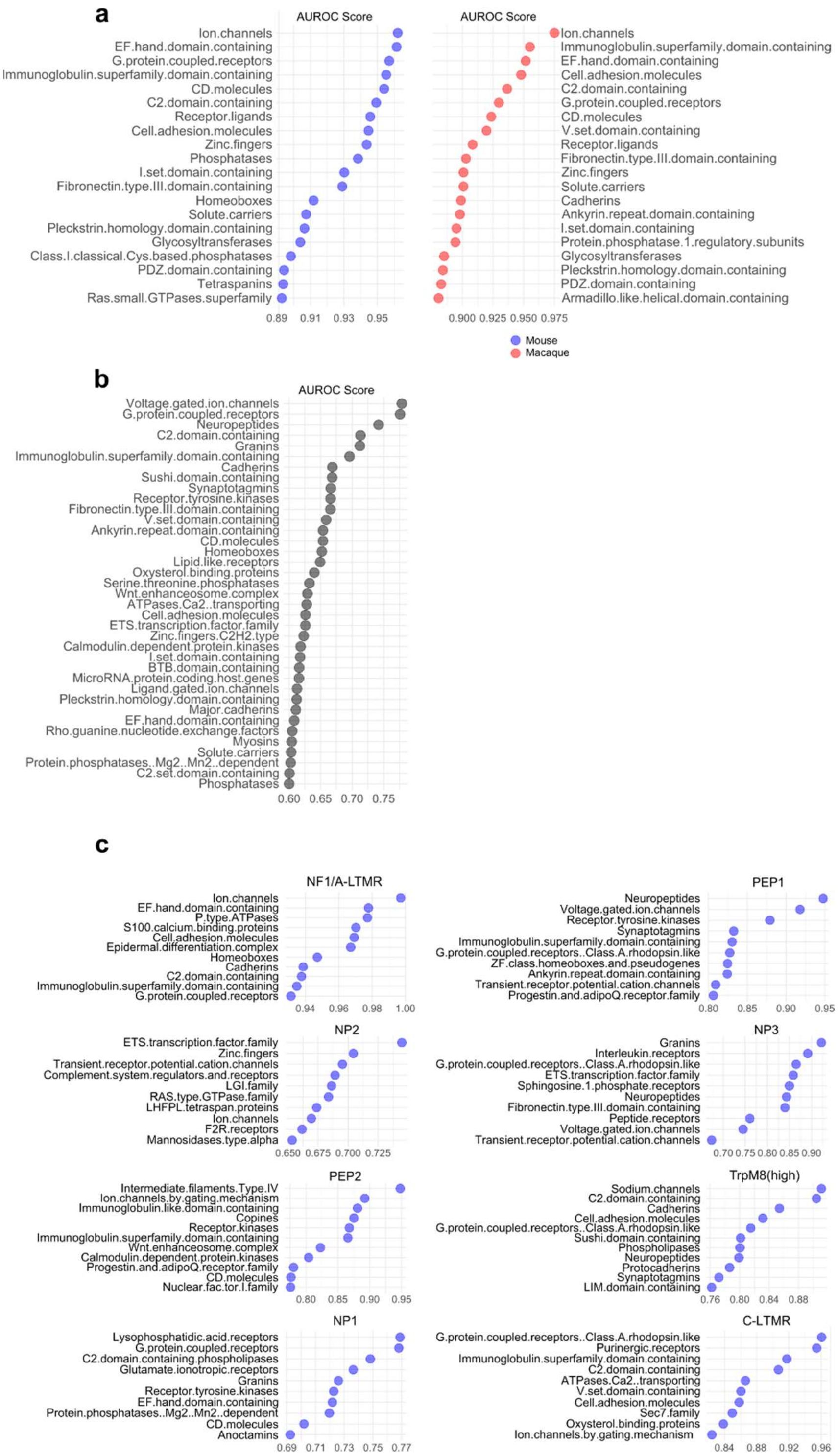

Supplementary Fig. 8. Computational screen of gene families that define DRG cell types. (a) Gene families showing high performance in correctly assigning cell types in both macaque and mouse. X-axis shows the average AUROC score over all cell types. (b) Gene families showing the highest performance in correctly assigning cell types between corresponding cell types of macaque and mouse. X-axis shows the average AUROC score over all cell types. (c) Gene families showing the highest performance in correctly assigning cell types between specific corresponding cell types of macaque and mouse. Note that the lists in the figure have been curated to remove highly redundant families. The full lists are available in Supplementary Tables 5 through 7.

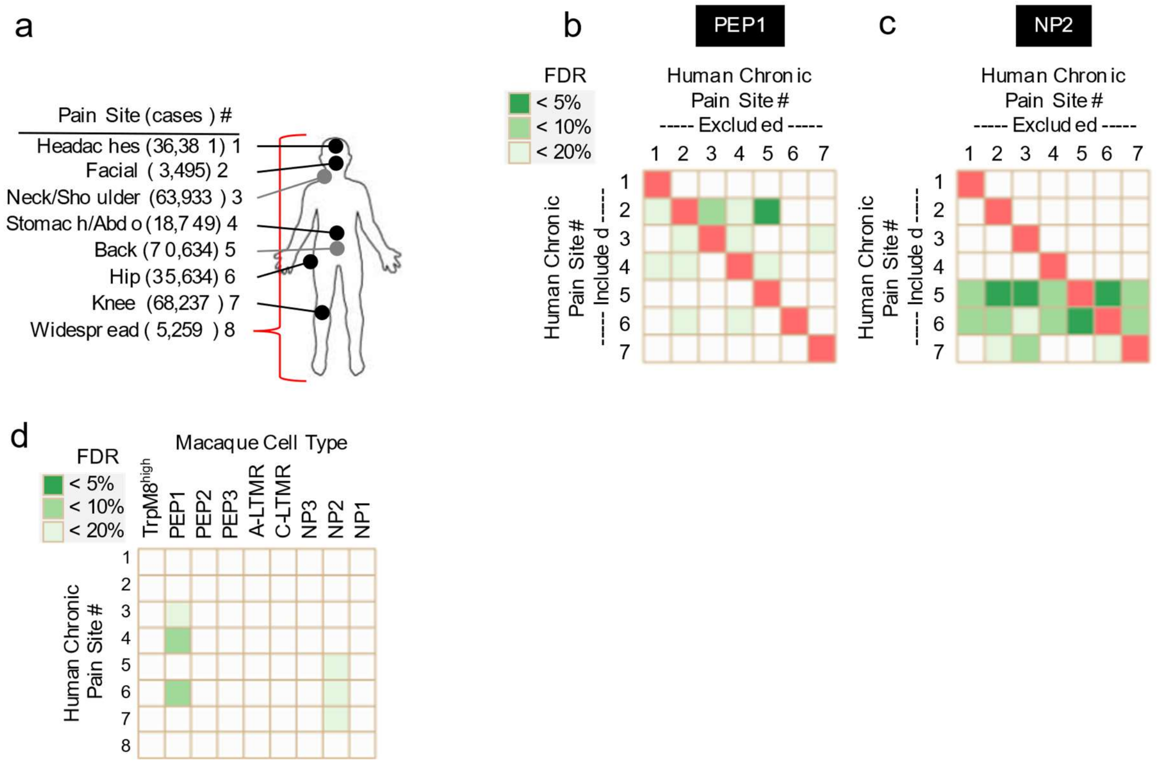

Supplementary Fig. 9. Leave-one-out analysis of partitioned heritability in the STRT-2i-seq data and partitioned heritability analysis in the Smart-seq2 data. (a) UK Biobank chronic pain sites mapped to the human body. Number of chronic case participants shown in parentheses. (b, c) Leave-one-out analysis of STRT-2i-seq data. Heatmaps of FDR-corrected p-values for enrichment in partitioned heritability of a macaque DRG cell type to each human chronic pain site (n=3 macaques). For a particular chronic pain site GWAS (row), the cases reporting other chronic pain site (column) were removed from the that GWAS. Widespread pain cases do not overlap with any other cases for the other sites (mutually exclusive questionnaire choices). (d) Partitioned heritability analysis in the Smart-seq2 data. Heatmap of FDR-corrected p-values for enrichment in partitioned heritability of each macaque DRG cell type to each human chronic pain site in the Smart-seq2 data (n=5 macaques). Image sources: wikipedia.org (human silhouette).
