# Supplementary material for: Single cell transcriptomics of primate sensory neurons identifies cell types associated with chronic pain
Source: Nat Commun. 2021 Mar 8;12:1510. doi: 10.1038/s41467-021-21725-z (PMC7940623; doi:10.1038/s41467-021-21725-z)
Supplement: Supplementary file 2 — Description of Additional Supplementary Files [file 41467_2021_21725_MOESM2_ESM.pdf]

Description of Additional Supplementary Files

Title: Supplementary Table 1.

Description: Marker genes for macaque DRG neuron types based on STRT-2i-seq data.

Title: Supplementary Table 2.

Description: Marker genes for macaque DRG neuron types based on Smart-Seq2 data.

Title: Supplementary Table 3.

Description: Comparison of marker gene expression across species.

Title: Supplementary Table 4.

Description: Gene families used in the MetaNeighbor analyses.

Title: Supplementary Table 5.

Description: Metaneighbor analysis results for macaque.

Title: Supplementary Table 6.

Description: Metaneighbor analysis results for mouse.

Title: Supplementary Table 7.

Description: Cross-species Metaneighbor analysis results.

Title: Supplementary Table 8.

Description: SCENIC regulons for macaque neuron types, STRT2i-seq data.

Title: Supplementary Table 9.

Description: SCENIC regulons defining mouse neuron types, Zeisel data

Title: Supplementary Table 10.

Description: GWAS and heritability of chronic pain.

Title: Supplementary Table 11.

Description: A full list of animals used in this study.

Title: Supplementary Table 12.

Description: Corresponding gene names between macaque and human.
