# Supplementary material for: Single cell transcriptomics of primate sensory neurons identifies cell types associated with chronic pain
Source: Nat Commun. 2021 Mar 8;12:1510. doi: 10.1038/s41467-021-21725-z (PMC7940623; doi:10.1038/s41467-021-21725-z)
Supplement: Supplementary file 15 — Reporting Summary [file 41467_2021_21725_MOESM15_ESM.pdf]

nature research

Corresponding author(s): Patrik Ernfors, Luda Diatchenko

Last updated by author(s): Jan 12, 2021

Reporting Summary

Nature Research wishes to improve the reproducibility of the work that we publish. This form provides structure for consistency and transparency in reporting. For further information on Nature Research policies, see our Editorial Policies and the Editorial Policy Checklist.

Statistics

For all statistical analyses, confirm that the following items are present in the figure legend, table legend, main text, or Methods section.

n/a	Confirmed	
☐	☒	The exact sample size (*n*) for each experimental group/condition, given as a discrete number and unit of measurement
☐	☒	A statement on whether measurements were taken from distinct samples or whether the same sample was measured repeatedly
☐	☒	The statistical test(s) used AND whether they are one- or two-sided<br>*Only common tests should be described solely by name; describe more complex techniques in the Methods section.*
☐	☒	A description of all covariates tested
☐	☒	A description of any assumptions or corrections, such as tests of normality and adjustment for multiple comparisons
☐	☒	A full description of the statistical parameters including central tendency (e.g. means) or other basic estimates (e.g. regression coefficient) AND variation (e.g. standard deviation) or associated estimates of uncertainty (e.g. confidence intervals)
☐	☒	For null hypothesis testing, the test statistic (e.g. *F*, *t*, *r*) with confidence intervals, effect sizes, degrees of freedom and *P* value noted<br>*Give P values as exact values whenever suitable.*
☒	☐	For Bayesian analysis, information on the choice of priors and Markov chain Monte Carlo settings
☒	☐	For hierarchical and complex designs, identification of the appropriate level for tests and full reporting of outcomes
☐	☒	Estimates of effect sizes (e.g. Cohen's *d*, Pearson's *r*), indicating how they were calculated

Our web collection on statistics for biologists contains articles on many of the points above.

Software and code

Policy information about availability of computer code

Data collection	No software was used in data collection.
Data analysis	R (v. 4.02), Python (v.3.8), ImageJ (v.1.52h), Seurat (v.3.2.2), METAL(2020-05-05), MAGMA(v.1.08), ldse(v1.01), LiftOver(v.3.12), MetaNeighbor(2018-03-01), SCENIC(v.1.1.2)<br>All analyses were based on previously published code (see below).<br>All code is available from the authors.<br>https://satijalab.org/seurat/v3.2/immune_alignment.html<br>https://satijalab.org/seurat/v3.2/integration.html<br>https://github.com/kharchenkolab/conos/blob/master/vignettes/walkthrough.md<br>https://github.com/linnarsson-lab/ipynb-lamanno2016/tree/master/scoringtool<br>https://github.com/linnarsson-lab/ipynb-lamanno2016<br>https://rawcdn.githack.com/aertslab/SCENIC/6aed5ef0b0386a87982ba4cc7aa13db0444263a6/inst/doc/SCENIC_Running.html<br>https://github.com/maggiecrow/MetaNeighbor<br>https://github.com/bulik/ldsc/wiki/LD-Score-Estimation-Tutorial<br>https://github.com/statgen/METAL<br>https://ctg.cncr.nl/software/magma

For manuscripts utilizing custom algorithms or software that are central to the research but not yet described in published literature, software must be made available to editors and reviewers. We strongly encourage code deposition in a community repository (e.g. GitHub). See the Nature Research guidelines for submitting code & software for further information.

Data

Policy information about availability of data

All manuscripts must include a data availability statement. This statement should provide the following information, where applicable:

- Accession codes, unique identifiers, or web links for publicly available datasets
- A list of figures that have associated raw data
- A description of any restrictions on data availability

GEO accession code for data generated in this study: GSE148238 (https://www.ncbi.nlm.nih.gov/geo/query/acc.cgi?acc=GSE148238)
Source data for figure 4b, c
Interactive web-application for the scRNA-seq data (https://ernforsgroup.shinyapps.io/macaquedrg/)
Macaca mulatta genome build (Mmul_10 / rheMac10, ftp://hgdownload.soe.ucsc.edu/goldenPath/rheMac10/)
Mouse DRG data (Zeisel): http://loom.linnarssonlab.org/clone/Mousebrain.org.level6/L6_Peripheral_sensory_neurons.loom
Mouse DRG data (Sharma): https://www.ncbi.nlm.nih.gov/geo/query/acc.cgi?acc=GSE139088

Field-specific reporting

Please select the one below that is the best fit for your research. If you are not sure, read the appropriate sections before making your selection.

☒ Life sciences ☐ Behavioural & social sciences ☐ Ecological, evolutionary & environmental sciences

For a reference copy of the document with all sections, see nature.com/documents/nr-reporting-summary-flat.pdf

Life sciences study design

All studies must disclose on these points even when the disclosure is negative.

Sample size	No sample size calculation was performed to pre-determine sample sizes.<br><br>For the scRNA-seq experiments we used sample sizes of n=3 (STRT-2i-seq)and n=5 (Smart-seq2); these sample sizes were ultimately restricted by the availability of animals; however, for scRNA-seq studies on cell type charcaterization these numbers of samples are sufficient for obtaining the necessary number of cells to perform a confident data analysis and characterization of cell types. The full reproduction of our results with two independent datasets (total n=8) validates the adequacy of our sample sizes in this study.<br><br>For the RNAScope validation tissue was derived from two animals. Because the celltype specific marker expression in individuals of the same species will be highly conserved with minimal variablility between individuals, this number of samples is enough for validations of the scRNA-seq cell types.<br><br>For GWAS in the UK Biobank, the sample size was determined by the number of available participants once genotyping quality controls have been performed followed by our own study exclusion criteria (described below). GWAS genotyping quality controls have been performed by the UK Biobank themselves, and are described in this document: https://biobank.ctsu.ox.ac.uk/crystal/crystal/docs/genotyping_qc.pdf.
Data exclusions	For scRNA-Seq data, we excluded low quality and non-neuronal cells through our quality control pipeline, as indicated in the methods section in the paper. In short, data was excluded based on thresholding of cells expressing less than the threshold set for counts and/or genes. Low quality, injured, non-neuronal and ambiguous cells (lacking specific marker genes) were omitted to enhance the quality of analysis.<br><br>GWAS participants were excluded based on: 1) poor genotyping quality, 2) voluntary retraction from the UK Biobank study, 3) of ancestries other than Caucasian (UK Biobank field 22006), 4) participants who answered “prefer not to answer” for the touchscreen question “In the last month have you experienced any of the following that interfered with your usual activities?” (UK Biobank field 6159).
Replication	The scRNA-seq studies were independently and successfully replicated using two different sequencing platforms (STRT-2i and Smart-seq2) on two different sets of animals (n=3 and n=5). Verification of the experimental findings derived from scRNA-seq was successfully performed through validation by RNAScope.<br><br>For GWAS, no other GWAS cohorts with the same recruitment characteristics (cross sectional) and matching pain questionnaires (self-declared at eight body sites, pain experienced for more than 3 months) were available for replication. The UK Biobank is one of the largest cohorts available for genetics studies. The UK Biobank cohort was used as a discovery cohort to test the hypothesis of involvement of genetic loci in genes expressed in specific cell types in primate dorsal root ganglia in pain phenotypes. For this, GWAS in sensory neurons was replicated using two independent datasets of macaque DRG neurons (STRT-2i n=3 and Smart-seq2 n=5 animals).
Randomization	The individual probe combinations for RNAScope validations were assigned to tissue slides randomly. No randomization of subjects in the UK Biobank for GWAS purposes was needed. For the cell-type specific partitioned heritability analysis size factors and animal’s sex were used as co-variables.
Blinding	RNAScope validation was done by a person without information of the underlying scRNA-seq cluster structure. Human genetics related bioinformatics was done without information related to the underlying scRNA-seq cluster structure.<br><br>The UK Biobank study was cross sectional in nature, and so is agnostic to self-declared pain status in participants. We did not do any

recruitment as part of the UK Biobank project. Study protocols were approved by UK Biobank's own "Ethics Advisory Committee". We analyze the data granted by UK biobank application number 20802.

Reporting for specific materials, systems and methods

We require information from authors about some types of materials, experimental systems and methods used in many studies. Here, indicate whether each material, system or method listed is relevant to your study. If you are not sure if a list item applies to your research, read the appropriate section before selecting a response.

Materials & experimental systems

n/a	Involved in the study
☒	☐ Antibodies
☒	☐ Eukaryotic cell lines
☒	☐ Palaeontology and archaeology
☐	☒ Animals and other organisms
☒	☐ Human research participants
☒	☐ Clinical data
☒	☐ Dual use research of concern

Methods

n/a	Involved in the study
☒	☐ ChIP-seq
☒	☐ Flow cytometry
☒	☐ MRI-based neuroimaging

Animals and other organisms

Policy information about studies involving animals; ARRIVE guidelines recommended for reporting animal research

Laboratory animals	8 rhesus macaques (Macaca mulatta), female and male, 5-15 years old.
Wild animals	No wild animals were used in the study.
Field-collected samples	No field-collected samples were used in the study.
Ethics oversight	The animal material was obtained from already euthanized animals sacrificed as a part of an unrelated study approved by the Stockholm Ethical Committee on Animal Experiments, organized under the Swedish Board of Agriculture (permit N2/15).

Note that full information on the approval of the study protocol must also be provided in the manuscript.
